# Acute Exercise and Neurocognitive Development in Preadolescents and Young Adults: An ERP Study

**DOI:** 10.1155/2017/2631909

**Published:** 2017-09-25

**Authors:** Chien-Heng Chu, Arthur F. Kramer, Tai-Fen Song, Chih-Han Wu, Tsung-Min Hung, Yu-Kai Chang

**Affiliations:** ^1^Graduate Institute of Athletics and Coaching Science, National Taiwan Sport University, Taoyuan, Taiwan; ^2^Department of Psychology, Northeastern University, Boston, MA, USA; ^3^Beckman Institute, University of Illinois, Urbana-Champaign, Champaign, IL, USA; ^4^Department of Physical Education, National Taiwan Normal University, Taipei, Taiwan

## Abstract

The purpose of this study was to examine the effect of a single bout of exercise on neurocognitive function in preadolescent children and young adults by determining the modulatory role of age and the neuroelectrical mechanism(s) underlying the association between acute exercise and executive function. Twenty preadolescents and 20 young adults completed the Stroop test, and neuroelectrical activity was recorded during two treatment sessions performed in a counterbalanced order. Exercise treatments involved moderate intensity aerobic exercise for 20 min as the main exercise and two 5 min periods of warm-up and cool-down. The control treatment participants read for a similar duration of time. Acute exercise improved participant reaction times on the Stroop test, regardless of Stroop congruency, and greater beneficial effects were observed in young adults compared to those in preadolescents. The P3 amplitudes increased after acute exercise in preadolescents and young adults, but acute exercise induced lower conflict sustained potential (conflict SP) amplitudes in preadolescent children. Based on these findings, age influences the beneficial effect of acute exercise on cognitive performance in general. Furthermore, the event-related brain potential differences attributed to acute exercise provide a potential clue to the mechanisms that differentiate the effects of acute exercise on individuals from preadolescence to young adulthood.

## 1. Introduction

The positive associations between exercise and a variety of psychological health outcomes, including reductions in anxiety and depression and improvements in emotion and mood, are well documented [1]. The beneficial effects of exercise on psychological health extend to cognition [2, 3], and even a single bout of aerobic exercise (i.e., acute exercise) has consistently been shown to positively influence cognitive function [4]. Specifically, the facilitation of cognitive performance by acute exercise of moderate intensity for 20 to 30 min has been reported in empirical studies (e.g., [5, 6]), qualitative reviews [7–9], and meta-analytical reviews [4, 10–12]. Notably, acute exercise is associated with improvements in a wide range of cognitive functions, including basic information processing, attention, crystallized intelligence, and executive function [4, 10], but a disproportionately larger benefit is observed for cognition related to executive function [13]. Studies of acute exercise and executive function typically emphasize effects on younger and/or older adult populations [5, 14–16], and only a few studies examined preadolescent children in a narrow age range (e.g., 9 to 10 years) [17, 18]. This research gap in children has generated several unanswered questions regarding executive function across childhood and adolescence [19] and how acute exercise affects executive function from the developmental perspective; therefore, this issue is worthy of further investigation [20, 21].

Executive function is an essential cognitive process; this function includes a number of components of higher level cognition, and it controls and regulates other more basic cognitive processes to achieve purposeful or goal-directed behaviors [22, 23]. Executive function has also been shown to determine the appropriate decision in response to nonroutine or conflict situations [24, 25]. Maturation of executive function occurs during early childhood and continues during young adulthood [26]. For example, Velanova et al. [27] observed better executive function performance in young adults (older than 17 years), followed by adolescents (aged 13 to 17 years), and then children (aged 8 to 12 years). The developmental trend in executive function parallels the neuroanatomical changes in brain regions associated with executive function [28]. According to functional magnetic resonance imaging (fMRI) research, children exhibit higher activation in the anterior cingulate cortex (ACC) and right dorsolateral prefrontal cortex (DLPFC) than adolescents and adults, suggesting that children engage more effort for a given task. Adolescents also exhibit more behavioral errors [27]. Based on these findings, neurocognitive development appears to be incomplete in preadolescents, and the maturation of this circuitry continues into adulthood [27].

Executive function constitutes distinct and multifaceted subcognitive processes, including the inhibition of prepotent responses, shifting between multiple sets, and updating working memory [29]. Previous acute exercise studies in healthy children predominantly explored the inhibitory aspect of executive function [17, 30, 31], and no consensus has been reached. For example, acute exercise facilitated inhibitory performance in some studies [30, 31] but failed to influence inhibition in other studies [17, 32]. Inhibition is associated with academic achievement [33], analogical reasoning [34], and emotional regulation [35] and is therefore particularly important in children.

Researchers have not yet conclusively determined whether age moderates the effect of acute exercise on executive function. According to meta-analytic reviews, acute exercise positively influences both high school-aged and young adults (i.e., 14 to 30 years) but not elementary-aged children (i.e., 6 to 13 years) [4]; however, the positive effect elicited by acute exercise was not different among preadolescents, adolescents, and young adults [36]. As shown in the study by Best [21], both age and the nature of executive function may be moderated by the relationship between acute exercise and executive function. For instance, prior research indicated significant influence of acute exercise on task-switching performance among young adults [37, 38], but not among children [39]. On the other hand, improved performance in the flanker task was reported among fit children [40], but not among young adults [41, 42]. However, to date, no acute exercise study has simultaneously examined inhibitory function among different age groups. Because the maturation of several cortex regions (i.e., ACC and DLPFC) occurs during neurocognitive development [27, 28, 43], as well as the association between neurotrophic factors (e.g., serum brain-derived neurotrophic factor, which plays a significant role in executive function) and increased brain volume [27, 28], age might differentially impact the cognitive performance of children and young adults in response to acute exercise. As a result, more research on the effects of acute exercise on inhibition in children and across the age spectrum is required to improve our understanding of this topic.

Exercise-induced arousal has frequently been proposed as a potential mechanism for the beneficial effect of acute exercise on executive function [10]. Specifically, the inverted U-trend of the arousal-performance relationship indicates that the optimal effect of acute exercise on cognitive performance is obtained when arousal is induced by moderate intensity exercise [4, 9, 10, 12]. Studies utilizing electrophysiological techniques, such as event-related potentials (ERPs), have provided additional insights into the mechanisms connecting acute exercise and cognition. An ERP is the pattern of neuroelectrical activation in response to, or in preparation for, an event (e.g., a stimulus). ERPs are recorded with high temporal resolution and reflect distinct cognitive processing between stimulus engagement and response execution [44]. P3 or the P300 component is an endogenous and positive stimulus-locked ERP component that occurs approximately 300 to 800 ms after a deviant event (e.g., a stimulus), and the maximal amplitude of P3 is observed over parietal electrode sites [45]. The P3 amplitude reflects the allocation of attentional resources during stimulus engagement [31, 45] and the level of physiological arousal [46]. P3 is also linked to developmental age, as an increased P3 amplitude has been documented to follow a maturational path from young children to adolescent children, reaches a peak value at approximately the age of 20, and then gradually declines with age [47].

Empirical studies have examined variations in P3 associated with acute exercise and observed that an increased P3 amplitude corresponded with improved behavioral performance, particularly for tasks that required inhibition following an acute bout of exercise [17, 31, 48]. Based on these findings, acute bouts of exercise benefit inhibitory performance by increasing the allocation of attentional resources during task performance. However, most previous studies of the associations of acute exercise with inhibition relied exclusively on P3, and fewer efforts have been focused on other ERP components. The current study examined P3 and the conflict sustained potential (conflict SP) component, which is a tonic, sustained, and conflict-sensitive slow potential that is frequently observed in the Stroop Task [49–51]. The polarity of the conflict SP is a region-dependent component that occurs approximately 500 ms after stimulus onset, with greater positivity over the central-parietal region and greater negativity over frontal regions following incongruent trials than after congruent trials [50, 52]. The conflict SP over the central-parietal regions likely reflects neural activity that responds to the presence of conflict [50, 52] or response selection [53]. Notably, the conflict SP during the Stroop test is more sensitive to conflict than P3 [54, 55], suggesting that this component is appropriate for examining the neurocognitive effects of acute exercise in the current study.

The current study examined the effects of acute exercise on the neurocognitive function of preadolescents and adults using the Stroop test. Specifically, the influence of age on the effect of acute exercise on executive function was elucidated. Furthermore, the neuroelectrical measures P3 and conflict SP were investigated to examine the potential mechanisms connecting acute exercise to neurocognitive function. We hypothesized that acute exercise would facilitate interference suppression in both populations, but young adults would show a larger beneficial effect. Similar patterns of larger P3 and conflict SP amplitudes would also be expected following the acute exercise intervention, with young adults exhibiting greater activation, suggesting that acute exercise differentially impacts cognitive function during the different stages of neurocognitive development.

## 2. Methods

### 2.1. Participants

Forty participants (*n* = 20 preadolescent male children; *n* = 20 young adults, 18 males and two females) were recruited through flyers posted in primary schools or universities in Taoyuan County, Taiwan. All participants were initially screened using the Physical Activity Readiness Questionnaire (PAR-Q) and Health Screening Questionnaire (HSQ) to ensure their safety prior to engaging in our fitness test and a single bout of moderate exercise [56]. Participants were also required to meet the following inclusion criteria: (a) right-handedness; (b) no history of psychological disorders, psychosis, neurological disorders, or head trauma; (c) no first-degree relatives with a history of psychosis; (d) not currently using any medications that may affect central nervous system function or cognitive performance; and (e) normal or corrected-to-normal vision and normal color vision. Demographic measures (e.g., age, body mass index, and education level) and working memory, which may influence performance on the Stroop test, were assessed [57]. Specifically, the Digit Span Forwards and Backwards tests of the Wechsler Adult Intelligence Scale-Third Edition (WAIS-III) and Wechsler Intelligence Scale for Children (WISC-R) [58] were administered to young adults and preadolescents, respectively. These tests have high test-retest reliability [59]. Participants and their legal guardians provided written informed consent, as indicated in the study protocol approved by the National Taiwan Sport University committee for institutional review.  Table 1 presents the demographic data for the young adult and preadolescent groups.

### 2.2. Cardiovascular Fitness Assessment

The cardiovascular fitness of the preadolescents and young adults was estimated using the single-stage submaximal treadmill walking test (SSTWT) developed by Ebbeling et al. [60]. The SSTWT is a convenient protocol for estimating the VO_2_ peak (VO_2peak_) in individuals of various ages and fitness levels [61], and it has been used previously [61–63]. The SSTWT includes two 4 min stages. Participants warmed-up on the treadmill at a comfortable speed between 2.0 and 4.5 mph at a 0% grade during the first 4 min stage, and the treadmill speed increased gradually until the participant's heart rate (HR) reached 60% to 70% of the maximum heart rate (HRmax). The estimated HRmax was calculated using the formula “208 − (0.78 × age)” for preadolescents [64, 65] and “207 − (0.67 × age)” for young adults [66, 67]. Following the 4 min warm-up stage, participants were asked to maintain the same speed for an additional 4 min while the treadmill inclined to 5%. The steady-state heart rate (SSHR) was recorded as the moment when the HR did not differ by more than 5 bpm during the final two minutes of the second 4 min period. VO_2peak_ was computed from the following formula: VO_2peak_ = 15.1 + 21.8 × speed (mph at 5% grade) − 0.327 × HR (bpm at 5% grade) − 0.263 – speed × age (year) + 0.00504 × HR × age + 5.96 × gender (0 = female; 1 = male).

### 2.3. Stroop Color-Word Test

A modified computerized Stroop color-word test (Stroop test) developed by Stroop [68] was used in this study. The Stroop test consists of congruent trials, in which three color words (i.e., red, blue, and green in Chinese) are presented in the ink of the color indicated by the word, and incongruent trials, in which the three color words are presented in ink of a nonmatching color. The three color words, each 2 cm^2^ in size, were displayed in the center of a 15-inch screen with horizontal and vertical angles of 28.14° and 1.40°, respectively, using the Neuroscan Stim2 software (Neurosoft Labs Inc., Sterling, VA, USA). The distance between the screen and participant was approximately 70 cm. Participants were required to complete 6 blocks of 60 trials, in which the congruent and incongruent trials in each block were arranged in a random order at a ratio of 2 : 1. The total length of the Stroop task was approximately 25 min. A fixed cross first appeared in the center of the screen for 506 ms, and stimuli were shown for 500 ms each. Participants were instructed to respond to the ink color according to the color on a response pane (10 × 8 × 2 cm box) by pressing one of the three colored buttons with their right thumb as quickly and accurately as possible. Responses were accepted between 200 ms and 1000 ms following the stimulus presentation. Responses outside the acceptable time window or with the wrong key were considered inaccurate responses. The reaction times and accuracies of the participants were recorded and analyzed as the primary indices.

### 2.4. ERP Assessment

Electroencephalography (EEG) was performed using an elastic cap (Quick-Cap, NeuroScan Inc., El Paso, TX, USA) with 32 Ag/AgCl electrodes that were mounted and arranged in accordance with the International 10–20 system [69]. All EEG recordings were referenced to the average of the right and left mastoid, and the ground electrode was placed on the AFz electrode site. The electrooculogram (EOG) activity was recorded from electrodes attached below and above the left eye (VEOG) and electrodes located at the outer canthi of both eyes (HEOG). Electrode impedance was maintained at or below 10 kΩ prior to testing. Continuous EEG data were amplified using a SynAmps EEG amplifier and the Scan 4.5 package (NeuroScan Inc., El Paso, TX, USA), with digitization at a 500 Hz sampling rate and an amplification of 500 times. A 60 Hz notch filter was also applied to remove potential artifacts.

Offline individual EEG data from correct trials were segmented into epochs from 200 ms prestimulus to 1000 ms poststimulus. Baseline correction was performed using the 100 ms period prior to stimulus onset, and the data were filtered using a 30 Hz zero phase shift (12 dB/octave, low-pass cutoff). The horizontal and vertical eye movement artifacts and blinks were corrected using Semlitsch et al.'s [70] algorithm. Amplitude excursions of ±100 *μ*V were rejected. The ERP waveform analysis focused on P3 and conflict SP. The final remaining correct trial numbers from the control and exercise conditions for both groups were recorded. P3 was calculated separately at the Fz, Cz, and Pz sites from the mean voltage from 300 to 450 ms after stimulus onset. The conflict SP component was quantified as the mean amplitude from 600 to 800 ms following stimulus onset, and the average conflict SP amplitudes of the right and left hemispheres were calculated separately (left central-parietal hemisphere: CP3 and P3; right central-parietal hemisphere: CP4 and P4).

### 2.5. Experimental Procedure

Participants visited the laboratory three times at least 24 hours apart at approximately the same time of day. The legal guardians of the preadolescents and the young adults were briefly introduced to the study during the first visit and completed the informed consent form, a demographic questionnaire, IPAQ, and PAR-Q to screen for inclusion status. Eligible participants were subjected to the SSTWT to estimate their cardiovascular fitness. Instructions and practice for the Stroop test (i.e., 15 trials) were given, and all participants reached the 85% accuracy rate before further assessment. This accuracy criterion was employed to limit the learning effect.

Participants attended one of the two treatments (control or exercise session) in a counterbalanced order on the second and third visits to eliminate any potential learning or practice effects. During the exercise session, aerobic exercise was performed on a motor-driven treadmill in a temperature-controlled room (mean temperature 22°C). The exercise protocol consisted of a 5 min warm-up phase, a 20 min main exercise phase, and a 5 min cool-down phase. Participants were instructed to run at 2.5 mph on a motor-driven treadmill that gradually increased in speed to reach the target 65–75% heart rate reserve (HRR) during the 20 min main exercise phase. This target HR range is considered moderate intensity, which is suggested to benefit cognitive performance [6, 9]. Participants were instructed to read educational documents for 30 min in a quiet room during the control session. Participants were escorted to an adjacent soundproof room immediately after each intervention to record the EEGs elicited during the Stroop test.

A polar HR monitor (Sport Tester PE 3000, Polar Electro Oy, Kempele, Finland) was utilized throughout the experimental procedure, and three HR indices were identified: resting HR, HR after 10 min of rest, and treatment HR, which was the average HR recorded during the 20 min exercise phase. The rating of perceived exertion (RPE) on the Borg scale [71] was recorded every 2 min during the exercise session. Participants received $40 and a brief review of the study purpose after completion of the experiment.

### 2.6. Data Analysis

The protocol employed a mixed design with Group as a between-subjects factor and the Treatment and Stroop congruency as within-subjects factors. Behavioral data (i.e., reaction time and accuracy) were analyzed using a three-way repeated-measures analysis of variance (ANOVA): 2 (Treatment: exercise and control) × 2 (Group: preadolescents and young adults) × 2 (Stroop congruency: congruent and incongruent). The remaining correct trial numbers were analyzed using a two-way repeated-measures ANOVA: 2 (Treatment) × 2 (Group). The mean averaged P3 amplitude was analyzed using a four-way repeated-measures ANOVA: 2 (Treatment) × 2 (Group) × 2 (Stroop congruency) × 3 (Site: Fz, Cz, and Pz), and the mean averaged conflict SP amplitude was analyzed using a different four-way ANOVA: 2 (Treatment) × 2 (Group) × 2 (Stroop congruency) × 2 (Site: averaged C3 and CP3 and averaged C4 and CP4). A Greenhouse-Geisser correction was used to adjust for family-wise error when the sphericity assumption was violated. The subsequent analyses consisted of univariate ANOVA and paired *t*-tests with Bonferroni's correction when appropriate. A partial eta-squared (*η*^2^) value for the effect size was reported and represented as small (i.e., 0.01 to 0.059), medium (i.e., 0.06 to 0.139), and large (i.e., >0.14) values [72]. SPSS versus 18 was used for the statistical analyses, and the significance level was set at alpha = 0.05.

## 3. Results

### 3.1. Exercise Manipulation Analysis

The average heart rates during the control session were 72.10 ± 9.94 and 68.67 ± 5.10 (bpm) for preadolescent children and young adults, respectively. Regarding the manipulation of the exercise intensity, the mean heart rates during exercise sessions were 157.77 ± 4.71 and 150.79 ± 7.65 (bpm) for preadolescent children and young adults, respectively; these values were within the range of 60% to 75% of the HRR. This range of HRR corresponds to the moderate intensity zone, suggesting that our procedure achieved an appropriate exercise intensity.

### 3.2. Behavioral Measures

#### 3.2.1. Reaction Time

**Figure 1:**
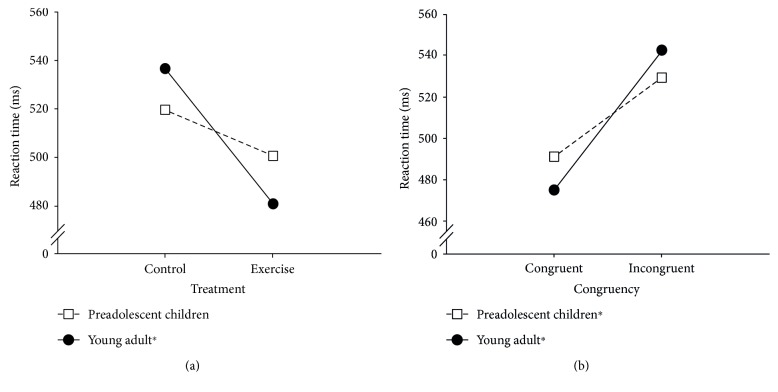
(a) Interaction effect of treatment and group. (b) Interaction effects of condition and group. ^∗^Significant difference (*p* < 0.05).

A three-way ANOVA revealed a main effect of Treatment, which was superseded by a Treatment × Group interaction (for detailed statistical values, see Table 2). Subsequent analyses revealed a significantly shorter reaction time in the exercise session compared to the control session for young adults (481.09 ms versus 536.57 ms), *F* (1, 19 = 21.13, *p* < 0.0001, *η*^2^ = 0.53). A marginally significantly shorter reaction time was observed in the exercise session compared to the control session for preadolescent children (500.80 ms versus 519.62 ms), *F* (1, 19) = 4.12, *p* = 0.057, *η*^2^ = 0.18. No differences were observed in the control session or the exercise session between preadolescents and young adults (Figure 1(a)).

A three-way ANOVA revealed a main effect of congruency, which was superseded by a Stroop congruency × Group interaction (Table 2). Subsequent analyses revealed a significantly longer reaction time in the incongruent condition than in congruent sessions for young adults (542.52 ms versus 475.14 ms), *F* (1, 19) = 674.83, *p* < 0.0001, *η*^2^ = 0.97 and for preadolescent children (529.33 ms versus 491.09 ms), *F* (1, 19) = 517.48, *p* < 0.0001, *η*^2^ = 0.97. No differences were observed in the congruent condition or the incongruent condition between preadolescents and young adults (Figure 1(b)). No other significant effects were observed.

#### 3.2.2. Accuracy

A three-way ANOVA revealed a main effect of Stroop congruency with higher accuracy in the congruent condition than the incongruent condition (87% versus 76%, *p* < 0.0001) (Table 2). A main effect of Group, which was superseded by a Treatment × Group interaction, did not reveal significant differences between preadolescents and young adults (78% versus 85%). No other significant effects were observed.

### 3.3. ERP Measurements

For the remaining correct trial numbers, the main effects of Treatment (exercise = 212.6 ± 54.36; control = 219.95 ± 29.98) or Group (preadolescents = 217.7 ± 27.68, young adults = 206.8 ± 50.93) or an interaction effect was not observed.

#### 3.3.1. Mean Averaged P3

**Figure 2:**
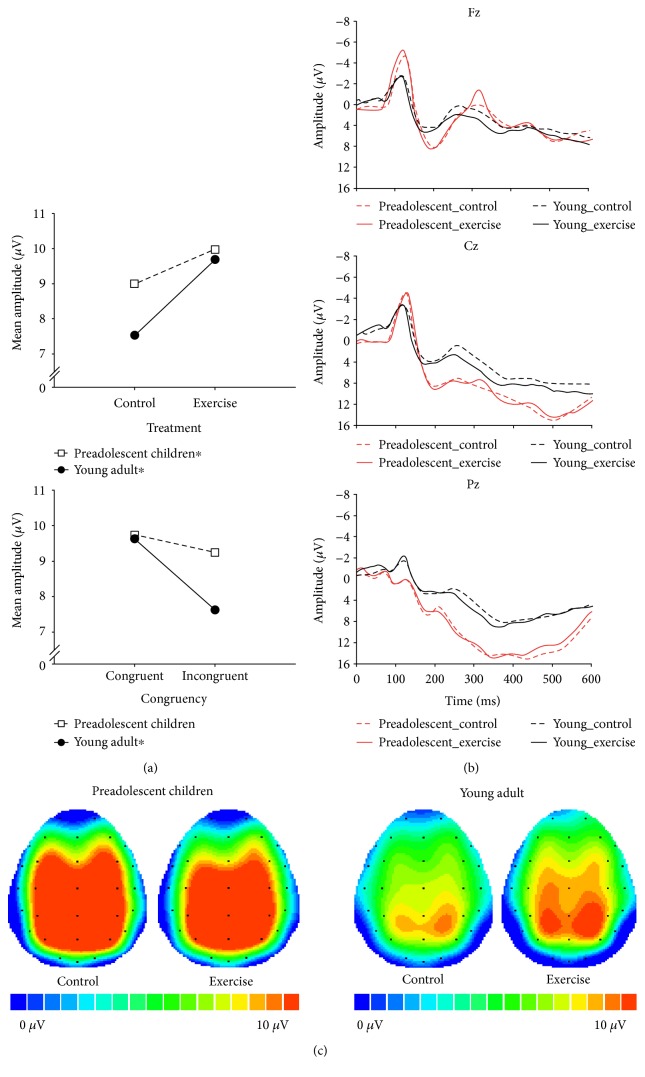
(a) Main effect of treatment and the interaction effect of congruency and group. (b) Stimulus-locked grand-average waveform at Fz, Cz, and Pz, collapsed across congruency in treatments and groups. (c) Topographic scalp distribution of the P3 amplitude collapsed across congruency in treatments and groups. ^∗^Significant difference within a group (*p* < 0.05).

A four-way ANOVA revealed a main effect of Treatment (Table 2), with larger P3 amplitudes in the exercise session than the control session (9.84 *μ*V versus 8.27 *μ*V, *p* = 0.04) (Figure 2(a)).

A main effect of Stroop congruency was superseded by a Stroop congruency × Group interaction. Subsequent analyses revealed a significantly smaller P3 amplitude in the incongruent trials than in the congruent trials for young adults (8.28 *μ*V versus 9.72 *μ*V, *p* = 0.02), but not for preadolescent children (9.86 *μ*V versus 10.81 *μ*V, *p* = 0.08) (Table 2). No differences were observed in the congruent and incongruent conditions between preadolescent and young adult individuals (Figure 2(b)).

A main effect of Site was superseded by a Site × Group interaction. Subsequent analyses revealed that the P3 amplitude was largest at Pz, followed by Cz and Fz, in preadolescent children (ps < 0.0001) and young adults (ps < 0.0001). Only P3 at Pz exhibited significant differences between groups (*p* = 0.02) (Table 2). The topographic distribution of the grand mean P3 amplitude across the scalp for each group and treatment is illustrated in Figure 2(c). No other significant effects were observed.

#### 3.3.2. Conflict SP

**Figure 3:**
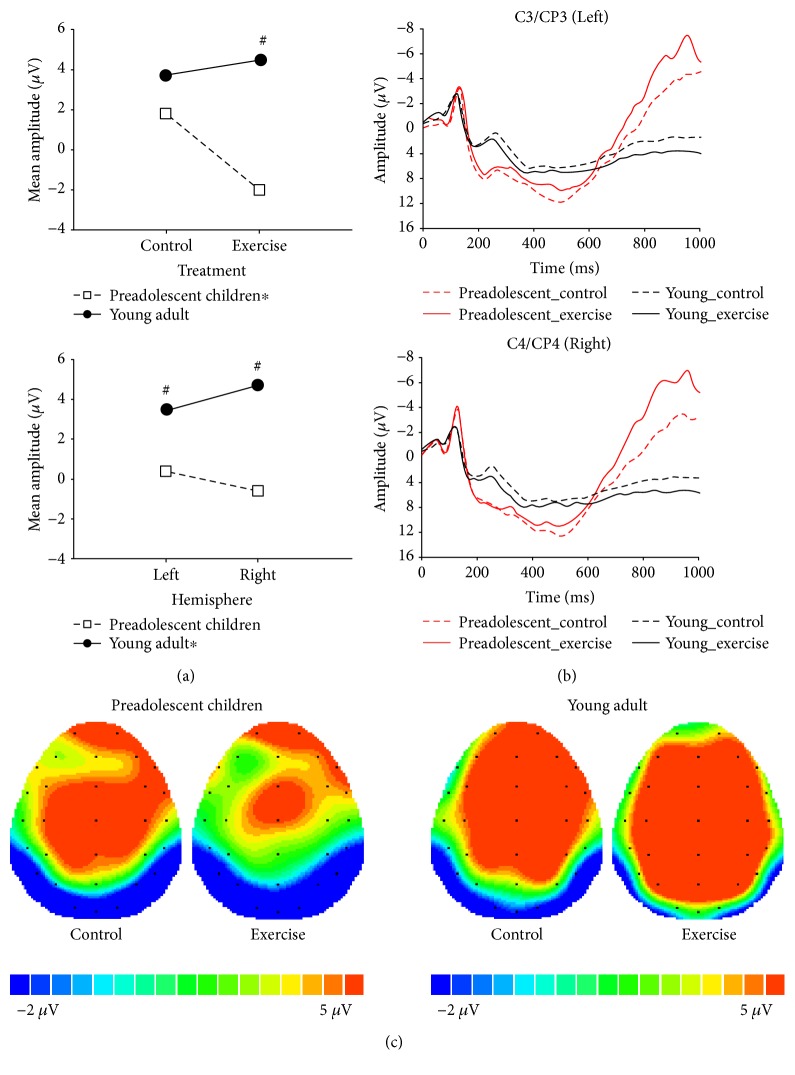
(a) Interaction effect of treatment and group and site and group. ^∗^Significant difference (*p* < 0.05). (b) Stimulus-locked grand-average waveform at the right and left central-parietal hemisphere collapsed across congruency in treatments and groups. (c) Topographic scalp distribution of the conflict SP amplitude collapsed across congruency in treatments and groups. ^∗^Significant difference within a group (*p* < 0.05); ^#^Significant difference between groups (*p* < 0.05).

A Treatment × Group interaction was observed (Table 2), and subsequent analyses revealed that the conflict SP amplitude was significantly smaller in the exercise session than that in the control session for preadolescents (−1.63 *μ*V versus 1.60 *μ*V, *p* = 0.03), but not for young adults (4.54 *μ*V versus 4.04 *μ*V, *p* = 0.51). Additionally, a difference in the conflict SP amplitude between preadolescent children and young adults was observed for the exercise session (*p* < 0.0001) but not the control session (*p* = 0.09) (Figure 3(a)).

A four-way ANOVA revealed a main effect of Stroop congruency, with larger conflict SP amplitudes in the incongruent condition than in the congruent condition (2.81 *μ*V versus 1.16 *μ*V, *p* < 0.001).

A Site × Group interaction was observed (Table 2), and subsequent analyses revealed a significantly larger conflict SP amplitude in the right hemisphere than in the left hemisphere in young adults (4.91 *μ*V versus 3.49 *μ*V, *p* = 0.004), but this interaction was not observed in preadolescent children (−0.48 *μ*V versus 0.30 *μ*V, *p* = 0.45). Additionally, a difference in the conflict SP amplitudes was observed between preadolescent children and young adults in the right hemisphere (*p* = 0.003) and left hemisphere (*p* = 0.0001) (Figure 3(b)). No other significant effects were observed. The topographic distribution of the grand mean conflict SP amplitude across the scalp for each group and treatment is illustrated in Figure 3(c).

## 4. Discussion

The current study extended the literature on acute exercise and cognition by investigating the modulatory role of age during preadolescence and adulthood using the behavioral and neuroelectrical indices of the Stroop test. Based on our primary findings, moderate intensity acute exercise for 20 min improved cognitive performance on the Stroop test for both Stroop congruency conditions, and these beneficial effects were greater in young adults than in children. Specifically, young adults exhibited improved performance in reaction time after the cessation of the acute exercise, but preadolescent children exhibited only marginally improved performance following exercise. Acute exercise had also differential effects on ERP indices in preadolescent children and young adults. Specifically, larger P3 amplitudes were observed in preadolescent children and young adults following acute exercise. No differences were observed in the conflict SP amplitudes between the two treatments in young adults, but the conflict SP amplitudes in preadolescent children were significantly reduced following acute exercise.

### 4.1. Acute Exercise and Behavioral Performance

Acute exercise improved the cognitive performance of young adults, regardless of Stroop congruency. Additionally, the longer reaction time and lower accuracy in incongruent trials compared to those in congruent trials reflected a robust “Stroop effect,” which is the response in incongruent trials which involves greater executive control because of competition between the stimulus-response translations that are introduced by task-relevant (i.e., ink color) and task-irrelevant (i.e., word meaning) stimuli [5, 13, 73, 74]. A selective improvement in the Stroop incongruent condition was reported after acute exercise [75], but our findings of improvements in the Stroop incongruent and congruent trials are partially consistent with recent studies in adults [5, 6, 13]. For example, Chang et al. [13] assessed the influence of acute exercise on five conditions of the Stroop test (i.e., congruent, word, square, neutral, and incongruent conditions) and observed that acute exercise had the largest positive effect on Stroop incongruent trials, but performance was enhanced for all five Stroop test conditions, which appears to reflect selective and general improvements. Alternatively, the enhanced behavioral performances induced by acute exercise may be due to more general effects on perception or response preparation. Although the statement may require further examination, acute exercise generally enhanced cognitive functions associated with the Stroop test in an adult population in our study.

Interestingly, the acute exercise-related improvements in Stroop test performance were significant in young adults, but only a positive trend was observed in preadolescent children. These results indicate a modulatory role of age on the interaction between acute exercise and cognition. Compared to young adults, children experienced a smaller beneficial effect on cognitive function after exercise cessation. Executive function and the brain are still developing in children [27, 76], which may render children less susceptible to changes elicited by acute exercise than young adults. However, our findings are inconsistent with previous studies that show facilitated interference suppression in the flanker task after acute exercise in preadolescent children, but the heterogeneous designs used in these studies should be considered. For example, acute exercise improved the response accuracy on a modified flanker task, but the behavioral index of reaction time [30, 31] and higher accuracy were observed only in children who displayed a lower inhibitory control capacity but not in children with a higher capacity [17]. Furthermore, studies [18, 48] also reported an acute exercise-induced improvement in inhibitory performance in children with attention-deficit/hyperactivity disorder (ADHD), which is linked to inhibitory dysfunction. Based on these findings, the beneficial effect of acute exercise on inhibition may be stronger in preadolescent children when inhibition is assessed using specific tasks and in children who are characterized by lower levels of or deficits in inhibitory capacity.

### 4.2. Acute Exercise and Neuroelectrical Activation

The larger P3 observed in adults and preadolescent children following exercise cessation is consistent with previous research using the flanker task in adults [77–79] and children [31, 48]. Our findings regarding acute exercise extended previous studies by revealing that neuroelectrical alterations might correspond to interference suppression during the Stroop test. The induction of a greater P3 amplitude by acute exercise regardless of Stroop congruency and age was also a novel finding. These results revealed a general rather than selective effect of acute exercise on the P3 amplitude for the different experiments in both age populations. The physiological arousal induced by acute exercise may be one of the primary mechanisms underlying improved cognitive function [80], and acute exercise-induced arousal and cognitive performance exhibit an inverted U-shaped correlation [77, 81–83]. Arousal induced by moderate intensity exercise leads to better performance compared to performances elicited by light or vigorous exercise. Arousal may be a potential mediator of the effects of acute exercise on cognition because of the positive relationship between arousal and P3 amplitude [45] and the similar inverted U-shape pattern for the correlation between exercise intensity and P3 amplitude [77, 81]. Additionally, the P3 amplitude is linked to the level of attentional allocation [45], and it is possible that our finding of a larger P3 amplitude after acute exercise might lead to an increase in arousal as well as in attention allocation, which might be used in the experimental task. Notably, acute exercise influenced cognitive processing in both age groups, as indicated by the neuroelectrical P3 index; this effect is unlike the modulatory role of age in the behavioral measurements, as preadolescent children received less of a positive effect of acute exercise than the benefit obtained by young adults. Thus, the effects of acute exercise are reflected by more sensitive indices at neuroelectrical levels, and these positive variations are similar in preadolescents and young adults.

Examination of the conflict SP provided another unique insight into the relationship between acute exercise and interference suppression and conflict resolution and conflict response selection. Young adults did not exhibit differences between the exercise and control sessions, and preadolescent children exhibited reduced conflict SP amplitudes following acute exercise, indicating that the modulatory role of age is illustrated by this specific ERP component. The current findings, which revealed a main effect of Stroop congruency on a greater conflict SP amplitude in incongruent trials than in congruent trials, are consistent with previous visual Stroop studies reporting that the conflict SP was proportional to the level of incongruence [50–52, 84]. Based on these results, the conflict SP reflects the cognitive resources that are recruited to resolve conflicts by selecting the proper response during the Stroop test [50, 85].

The maintenance of the conflict SP amplitudes between control and following acute exercise in young adults might indicate the lessened impact of acute exercise on conflict resolution or conflict response selection. Interestingly, prior research has suggested that acute exercise resulted in an increase in conflict detection indexed by the shorter N450 latency [86], suggesting the differential effects of acute exercise on the stages of conflict processing in young adults. In contrast to young adults, preadolescent children exhibit evidence of reduced conflict SP amplitude following acute exercise, which might be interpreted as a reduction in the interference effect and increased conflict-processing ability. This finding is accordance with a recent meta-analytic study [87] in which the authors suggested the greater benefits of acute exercise for preadolescent children who are undergoing executive function development changes, such as changes in the middle frontal gyrus and left extrastriate region [50].

### 4.3. Age and Stroop Congruency

The “Stroop effect” was observed in preadolescent children and young adults, with no differences between these two age groups. Our findings illustrated a robust interference effect that replicated the previous studies [5, 13, 73, 74] and indicated that the effect was similar in the groups with ages between 12 and 20 years. Inhibitory control dramatically increases in children between the ages of 3.5 and 5 years, and further improvements are only modest until 11 years of age [88]. Ikeda et al. [89] observed less Stroop interference in young adults than in 5- to 6-year-old, 7- to 8-year-old, and 9- to 10-year-old children. However, this difference was not observed when young adults were compared to 11- to 12-year-old children. Our findings are consistent with the reports showing that children aged approximately 12 years may have a similar inhibitory ability as young adults, but the behavioral measures may also have limited sensitivity to reflect age-specific differences. Our neuroelectrical indices may support this assertion.

Smaller P3 amplitudes were elicited in the incongruent condition than in the congruent condition in young adults in this study. The congruency-dependent P3 amplitude observed in young adults is consistent with the previous research [90–92]. Specifically, the smaller P3 amplitude is likely caused by the greater difficulty experienced in the evaluation and classification processes during incongruent trials [92]. Notably, the P3 amplitude did not reflect the Stroop congruency difference in preadolescent children. The P3 amplitude induced by visual stimuli likely decreases from childhood into early adulthood, as previously reported [93]. A few studies examined the conflict SP and Stroop congruency, and our finding of a main effect of Stroop congruency is consistent with the previous studies that observed larger conflict SP amplitudes in the incongruent condition. However, our study extended the idea of a greater conflict SP in both brain hemispheres in young adults than in preadolescent children. Based on these findings, young adults exhibit better interference suppression and conflict resolution abilities compared to those in preadolescent children. Moreover, young adults exhibited a larger conflict SP in the right central-parietal regions than that in the left regions; this finding replicates the results of a study of an applied conflict task that presented a Chinese stimulus [94]. Collectively, P3 neuroelectrical measures provide sensitive indices of the modulatory role of Stroop congruency and age during various developmental stages. More research is required to explore which specific ERP components are relevant during different stages of neurocognitive development.

### 4.4. Limitations and Future Directions

Certain limitations of the current study should be acknowledged and considered in future research. Despite the evidence that acute exercise improves interference suppression, as assessed by the Stroop test, a response recorded by key pressing cannot distinguish whether the acute exercise-induced facilitation results from the stimulus or response processing benefits [49, 95]. Specifically, the “Stroop effect” is produced by both stimulus-stimulus incompatible (e.g., the word “red” printed in blue with a verbal response, which results in semantic competition) and response-response incompatible relations (e.g., the word “red” printed in blue, with the pressing “blue” of the assigned buttons, which results in a response competition). Future research may use the Stroop test paradigm with two colors that are assigned to the same manual response [49] or a paradigm in which the incongruent trials are either incongruent-eligible or incongruent-ineligible [96] to further characterize the beneficial effect of acute exercise. Additionally, the interaction of acute exercise and cognition may be moderated by individual differences, such as cardiovascular fitness. For example, individuals with higher cardiovascular fitness exhibited superior cognitive performance following acute exercise compared to their counterparts with lower fitness [97]. Other factors that differ among individuals are education level and inhibitory control capacity. We did not observe differences in either the congruent or incongruent conditions between preadolescents and young adults in the control session, suggesting that all participants presented a similar reading ability for the easy words that were tested (i.e., red, green, and blue in Chinese characters). However, this finding also implies that the task may be insufficient at revealing the developmental differences in cognitive control mechanisms. A future study that considers education level and manipulates the degree of task difficulty is recommended. A third limitation may be related to the use of reading in the control session. The use of videogames may serve as a better “active control” protocol since it may be able to maintain the arousal levels of the participants and prevent them from becoming bored [98]. Additionally, although differences in gender were not observed across the two age groups (chi square = 2.11, *p* > 0.05), our unbalanced gender proportion, particularly more males than females, limits the interpretation and generalization of these findings. The current study did not examine the correlation between the acute exercise and behavioral and neurological indices; therefore, we cannot establish the mediating role of the neurological indices on the variation in behavioral performance. Future studies are encouraged to use larger numbers of participants and conduct mediation analysis to further establish the potential mediator function of P3 and conflict SP on behavioral improvement. Finally, the present study compared the periods of preadolescence and young adulthood, but the recruited preadolescent children were limited to ages between 10 and 12 years. According to a previous developmental research, changes in the inhibitory capacity differ dramatically across childhood [19], and interference suppression develops in a nonlinear pattern in children [89]. A better understanding of the effect of acute exercise on inhibitory control may be achieved by the inclusion of children across a wider age range and a longitudinal examination of executive function in children.

## 5. Conclusions

This study is the first to reveal that the beneficial effect of acute exercise on interference suppression in the Stroop test is moderated by age, with young adults experiencing more benefit than preadolescent children, who showed limited benefit from acute exercise. Young adults had a larger P3 amplitude and an unaffected conflict SP amplitude following acute exercise, but preadolescent children exhibited a larger P3 amplitude and reduced conflict SP amplitude, indicating divergent mechanisms from a neuroelectrical perspective. Although the beneficial effects of acute exercise on cognitive function may be attributed to more general effects on perception and response processes, improved cognitive performance may be associated with enhanced attentional allocation in both age populations, but the positive effects associated with interference suppression and conflict resolution were only observed in young adults. These findings extend the current knowledge base by revealing a modulatory role of age in the relationship between acute exercise and interference suppression and provide preliminary evidence for the potential underlying mechanism by which acute exercise positively affects interference suppression throughout early adulthood.

## Figures and Tables

**Table 1:** Participant demographic characteristics (mean ± 1 SD; range).

Variable	Group	
	Preadolescent children	Young adults
Sample size	20	20
Gender (female: male)	0 : 20	2 : 18
Age (yrs)	10.50 ±. 53; 10-11	20.42 ± 1.16; 19–23
Education (yrs)	4.40 ± .52; 4-5	14.33 ± 1.37; 13–18
Height (cm)	146.30 ± 8.79; 138.00–167.00	169.75 ± 5.82; 156.00–178.00
Weight (kg)	40.00 ± 6.13; 30.00–50.00	66.25 ± 10.80; 48.00–80.00
BMI (kg·m^−2^)	18.65 ± 2.14; 15.11–22.32	22.92 ± 3.13; 19.15–27.68
Digit span forward	14.30 ± 1.49; 11–16	14.50 ± 1.31; 12–16
Digit span backward	8.70 ± 2.40; 5–12	10.58 ± 2.97; 5–14
VO_2peak_ (mL·kg^−1^·min^−1^)	50.60 ± 8.24; 46.43–68.07	49.18 ± 7.57; 44.16–65.43
Resting heart rate	70.20 ± 6.78; 59.00–86.00	65.50 ± 5.27; 56.00–73.00

**Table 2:** Summary of statistical analyses of behavioral and ERP measures.

Measure and effect	df	*F*	*p*	*η* ^2^
*Stroop test reaction time*				
Treatment	1, 38	23.83	<.0001	.39
Treatment × group	1, 38	5.80	=.021	.13
Congruency	1, 38	60.65	<.0001	.62
Congruency × group	1, 38	4.62	=.038	.11
*Stroop test accuracy*				
Treatment × group	1, 38	7.03	=.012	.16
Congruency	1, 38	56.31	<.0001	.60
Group	1, 38	5.24	=.028	.12
*Mean averaged P3 amplitude*				
Treatment	1, 36	4.65	=.038	.11
Congruency	1, 36	24.66	<.0001	.41
Congruency × group	1, 36	9.02	=.005	.20
Site	2, 72	71.08	<.0001	.66
Site × group	2, 72	14.51	<.0001	.29
*Mean averaged SP amplitude*				
Treatment × group	1, 36	7.00	=.012	.16
Congruency	1, 36	13.23	=.001	.27
Site × group	1, 36	4.61	=.039	.11

*Note*. Only significant effects were presented.
